# The Annual Address to the Graduating Class in the Medical Institution of Yale College, January 15, 1852

**Published:** 1852-08

**Authors:** 


ART. VII.—*The Annual Address to the Graduating Class in the Medical Institution of Yale College, January* 15, 1852. By ALVAN TALCOTT, M. D., in behalf of the Board of Examiners.

This is an address full of sound and seasonable advice to a class of young gentlemen about to assume the duties of their profession. We do not know where they could go for better counsel than is comprised in the pages of this valedictory.

